# Histone lysine methyltransferases and their specific methylation marks show significant changes in mouse testes from young to older ages

**DOI:** 10.1007/s10522-025-10187-1

**Published:** 2025-01-20

**Authors:** Yesim Bilmez, Gunel Talibova, Betul Tire, Saffet Ozturk

**Affiliations:** Department of Histology and Embryology, Akdeniz University School of Medicine, Campus, 07070 Antalya, Türkiye

**Keywords:** Histone lysine methyltransferases, Histone methylation, Spermatogenesis, Male infertility, Testicular aging

## Abstract

Spermatogenesis is finely regulated by histone methylation, which is crucial for regulating gene expression and chromatin remodeling. Functional studies have demonstrated that the histone lysine methyltransferases (KMTs) SETD1B, CFP1, SETDB1, G9A, and SETD2 play pivotal roles in spermatogenesis through establishing the key histone methylation marks, H3K4me3, H3K9me2, H3K9me3, and H3K36me3, respectively. This study aimed to evaluate the spatiotemporal expression of these KMTs and methylation marks as well as senescence-associated β-galactosidase (β-GAL), transcriptional activity, and apoptosis rates in mouse testes during biological aging. In accordance with these purposes, the following groups of Balb/C mice were created: young (1- and 2-week-old), prepubertal (3- and 4-week-old), pubertal (5- and 6-week-old), postpubertal (16-, 18-, and 20-week-old), and aged (48-, 50-, and 52-week-old). The β-GAL staining gradually increased from the young to the aged groups (*P* < 0.01). The SETD1B, G9A, SETDB1, and SETD2 protein levels increased in spermatogonia, early and pachytene spermatocytes, and Sertoli cells of the aged group (*P* < 0.05). In contrast, CFP1 protein level decreased in spermatogonia, pachytene spermatocytes, round spermatids, and Sertoli cells towards the older ages (*P* < 0.05). Moreover, H3K4me3, H3K9me2, H3K9me3, and H3K36me3 levels increased in the aged group (*P* < 0.05). There was also a significant reduction in apoptosis rates in seminiferous tubules of the pubertal, postpubertal, and aged groups (*P* < 0.01). Consequently, accumulation of histone methylation marks due to increased expression of KMTs in spermatogenic and Sertoli cells during testicular aging may alter chromatin reprogramming and gene expression, contributing to age-related fertility loss.

## Introduction

Infertility represents a significant global health concern, with approximately 50% of cases attributed to male factors (Huang et al. 2023). Male infertility is a complex issue with a multitude of underlying causes, including genetic and environmental factors such as endocrine disorders, immunological problems, gene mutations, radiation, lifestyle, and aging (Campbell and Irvine 2000; Harris et al. 2011). The loss of male fertility associated with biological aging can be attributed to a number of factors, including decline in testicular function (Sharma et al. 2015), alterations in reproductive hormones (Feldman et al. 2002), deteriorations in sperm parameters (Brahem et al. 2011), DNA instability (Moskovtsev et al. 2006), telomere shortening (Broer et al. 2013), and epigenetic changes (Curley et al. 2011). The term "epigenetics" is used to describe alterations in gene expression that occur independently of changes in DNA sequence (Weinhold 2006). Histone methylation, one of the epigenetic mechanisms, plays a pivotal role in the spatiotemporal regulation of gene expression, which is necessary to maintain male fertility during mitotic and meiotic divisions as well as cellular differentiation in the spermatogenic cells during spermatogenesis (Güneş and Kulaç 2013).

The process of histone methylation involves a dynamic addition of methyl groups derived from S-adenosyl methionine (SAM) to histone tails, which are primarily appearing at lysine (K) or arginine (R) residues with rarely in histidine residues (Greer and Shi 2012). This process is initiated by histone methyltransferases (HMTs), which include protein arginine methyltransferases (PRMTs) and lysine methyltransferases (KMTs) based on the type of amino acid residues to which a methyl group is added (Nimura et al. 2010). Arginine residues can be methylated mono- (me1) and di- [me2; symmetrically (me2s) or asymmetrically (me2a)] (Greer and Shi 2012), whereas lysine residues can undergo me1, me2, or tri- (me3) methylation (Martin and Zhang 2005).

The KMTs, CFP1, SETDB1, G9A and SETD2, and their specific methylation marks, H3K4me3, H3K9me2, H3K9me3, and H3K36me3, are known to be involved in meiotic progression, homologous recombination, chromatin condensation, DNA repair, retrotransposon silencing and spermiogenesis through regulating gene expression during spermatogenesis (Bilmez and Ozturk 2023). These histone methylation marks have been identified in spermatogonia (Payne and Braun 2006), primary spermatocytes (Godmann et al. 2007), and round spermatids (Neto et al. 2016). These histone methylation marks show prominent distributions in spermatogenic cells, whose timely and correct establishment is critical for male fertility (Tahmasbpour Marzouni et al. 2022). Alterations in their accumulation can lead to reduced spermatocyte numbers, abnormal sperm morphology, increased apoptosis, impaired chromatin condensation, and male infertility [Reviewed in (Ge et al. 2017)].

CXXC1 finger protein 1 (CFP1) is a subcomponent of the SETD1B complex, which mediates formation of the transcriptional activation-related histone methylation H3K4me3 (Sugeedha et al. 2021). CFP1 is expressed in mouse spermatocytes and round spermatids but not in spermatogonia and elongated spermatids (Jiang, Zhang et al. 2020). Its absence leads to defective meiosis, disrupted spermatogenesis, and reduced H3K4me3 levels in spermatocytes (Jiang, Zhang et al. 2020). Tatehana *et al*., (2020) assessed the impact of biological aging on H3K4 methylation in testicular tissues obtained from young (3-month-old) and aged (12-month-old) mice (Tatehana et al. 2020). H3K4me3 was not detected in preleptotene, leptotene, zygotene, and pachytene spermatocytes, and a decrease was observed in round spermatids of the aged group. Consequently, transcription-related histone methylation (H3K4me3), predominantly generated by the action of CFP1, exhibits age-associated alterations in specific spermatogenic cells.

The G9A protein is involved in the catalysis of H3K9me2, which is indispensable for the progression of meiotic prophase in male germ cells (Tachibana et al. 2007). Conditional deletion of the *G9a* gene in male germ cells resulted in an increase in apoptosis, a reduction in H3K9me2 levels in spermatocytes, and male infertility. Additionally, G9A protein was identified in mouse spermatogonia and preleptotene spermatocytes (Tachibana et al. 2007). H3K9me2 displayed a diffused staining pattern in preleptotene, leptotene, and zygotene spermatocytes, whereas no H3K9me2 signal was observed in pachytene spermatocytes. As a result, H3K9me2 is subject to dynamic regulation during meiotic prophase, ensuring efficient germ cell development through the action of G9A.

The SET domain bifurcated histone lysine methyltransferase 1 (SETDB1) is responsible for generating H3K9me3 mark, which is associated with transcriptional repression (Barral et al. 2022). Conditional deletion of the *Setdb1* gene in male mouse germ cells has been observed to alter gene expression and disrupt the distribution of H3K9me3 in spermatocytes, ultimately resulting in male sterility (Cheng et al. 2021). SETDB1 was localized in spermatogonia, spermatocytes, round spermatids, Sertoli cells, and interstitial cells, with high H3K9me3 levels in undifferentiated spermatogonia in an adult pig testis (Liu et al. 2017a, b; Cheng et al. 2021). The process of biological aging is associated with a decline in the levels of H3K9me3 in a number of substages of the spermatogenic process, including preleptotene, leptotene, zygotene and pachytene spermatocytes, and round spermatids (Tatehana et al. 2020).

The transcriptional activation-related H3K36me3 is created by SET domain-containing protein 2 (SETD2) (Yuan et al. 2017). Conditional deletion of *Setd2* in mouse testis resulted in changes in expression of the genes involved in histone-protamine transition and spermiogenesis-linked events, and loss of H3K36me3 in the pachytene spermatocytes and round spermatids (Zuo et al. 2018). SETD2 was at high levels in pachytene spermatocytes, round spermatids, and Sertoli cells in the adult mouse testis. Consistent with SETD2 expression patterns, H3K36me3 reached high levels in all spermatocyte stages and round spermatids, and decreased to low levels in spermatogonia and elongated spermatids.

It is known that sperm concentration, motility, morphology, and testosterone levels decrease with biological aging, which result in male fertility loss (Harris et al. 2011; Khandwala et al. 2017; Collodel et al. 2021). However, molecular background of the mechanisms underlying loss of male fertility during paternal aging process has not yet been fully elucidated. In this study, we examine expression of the SETD1B, CFP1, G9A, SETDB1, and SETD2 proteins and the relative levels of H3K4me3, H3K9me2, H3K9me3, and H3K36me3 marks as well as transcriptional activity and apoptosis rates in the postnatal mouse testes from young to older ages.

## Materials and methods

### Animals and sample collection

In this study, we used male Balb/C mice categorized as young (1- and 2-week-old; n = 5 from each week), prepubertal (3- and 4-week-old; n = 4 from each week), pubertal (5- and 6-week-old; n = 4 from each week), postpubertal (16-, 18-, and 20-week-old; n = 4 from each week), and aged (48-, 50-, and 52-week-old; n = 6, 5, 6 from each week, respectively). We used paraffin blocks obtained from our previous study (Talibova et al. 2023), and the present study was conducted in accordance with a recent ethical approval that was provided by the Akdeniz University Institutional Animal Care and Use Committee (Protocol no. 1509/2022.09.005, Decision no. 116). Testis paraffin blocks were cut at 5 µm thickness and used for immunohistochemical staining, as described in detail below.

### Immunohistochemical staining

We performed immunohistochemical staining to determine spatiotemporal distribution and relative levels of the β-GAL, SETD1B, CFP1, G9A, SETDB1, and SETD2 proteins and the H3K4me3, H3K9me2, H3K9me3, and H3K36me3 marks in the postnatal testes as described in our previous studies (Bilmez et al. 2022; Talibova et al. 2023). In addition, immunostaining for pS2 and cCASP3 was performed to assess the transcriptional activity and apoptosis, respectively, which are target effects of the histone methylation marks.

Sections from the postnatal testes were placed in an oven at 60 °C for 1 h and deparaffinized in fresh xylene. Sections were treated with a graded series of alcohols for rehydration, and then antigen retrieval was performed by boiling sections in Tris–EDTA (containing 10 mM Tris base and 1 mM EDTA) for 15 min (10 min for H3K9me2 and H3K9me3 immunostaining) in a microwave set at 665 W. Endogenous peroxidase activity was then blocked by exposure to 3% H_2_O_2_ prepared in methanol for 25 min at room temperature (RT). After washing in 1XPBS, sections were incubated for 7 min in Ultra V blocking solution (catalog no. TP-060-HL, Thermo Scientific) to prevent non-specific binding of the secondary antibodies. After that sections were incubated overnight at + 4 °C with the appropriate antibodies as listed in Table 1.

**Table 1:** Primary antibodies used in immunohistochemical staining

Antibody target	Dilution of primary antibody	Manufacturer	Catalog no
β-GAL	1/100	Proteintech	11518-1-AP
SETD1B	1/1000	Proteintech	55005-1-AP
CFP1	1/1750	Abcam	ab198977
G9A	1/350	Bioss	bs-9118R
SETDB1	1/500	Bioss	bs-11670R
SETD2	1/500	Life Science	LS-C81092
H3K4me3	1/1000	Thermo Fisher Scientific	PA585525
H3K9me2	1/1000	Abcam	ab176882
H3K9me3	1/1000	Thermo Fisher Scientific	PA531910
H3K36me3	1/750	Thermo Fisher Scientific	PA596118
pS2	1/750	Thermo Fisher Scientific	MA532637
cCASP3	1/100	Cell Signaling	9661S

Critically, isotype IgG antibody (catalog no. I5006, Sigma-Aldrich) was used to stain negative control sections to determine specificity of the primary antibodies. The isotype antibody was diluted at the same concentrations as the primary antibodies. After primary antibody incubation, sections were washed in 1XPBS for 15 min. The sections were then treated with a biotinylated secondary antibody (goat anti-rabbit IgG, diluted 1:2000, catalog no. BA-1000, Vector Labs) for 1 h at RT, except for cCASP3 primary antibody. For cCASP3 primary antibody, horseradish peroxidase (HRP)-conjugated immunohistochemistry detection reagent (diluted 1:1, catalog no. 8114S, Cell Signaling) was used at RT for 1 h. The streptavidin-HRP complex (catalog no. TS-125-HR, Thermo Scientific) was then applied to the sections for 30 min at RT. Finally, immunoreactions were visualized with 3, 3′-diaminobenzidine (DAB) substrate (catalog no. D4168, Sigma-Aldrich) under a light microscope. We counterstained the sections with Mayer’s hematoxylin after washing under running tap water to visualize nuclei. Sections were dehydrated in graded alcohols, passed through xylene, and covered with a glass coverslip using Entellan.

ImageJ software [National Institutes of Health (NIH), Bethesda, Maryland, USA] was used to evaluate spatiotemporal distributions and relative levels of β-GAL, SETD1B, CFP1, G9A, SETDB1, SETD2, H3K4me3, H3K9me2, H3K9me3, H3K36me3, pS2, and cCASP3 in the postnatal testes from young to aged groups. This evaluation was performed for each seminiferous tubule, each germ and Sertoli cell. It is worth noting that we analyzed at least five images from each age for total analysis, ten seminiferous tubules, and twenty each spermatogenic germ and Sertoli cell types. Micrographs were taken at 200 × original magnification using a Zeiss Axiocam 105 color brightfield microscope. To calculate unit expression levels, the integrated means obtained from ImageJ software were divided into area values.

### Statistical analysis

One-way analysis of variance (ANOVA) on ranks and Tukey’s post hoc test were used to evaluate the data for statistical significance. GraphPad Prism 9 was used for all statistical calculations, with* P* < 0.05 considered statistically significant.

## Results

### β-GAL expression in the postnatal mouse testes

**Fig. 1:**
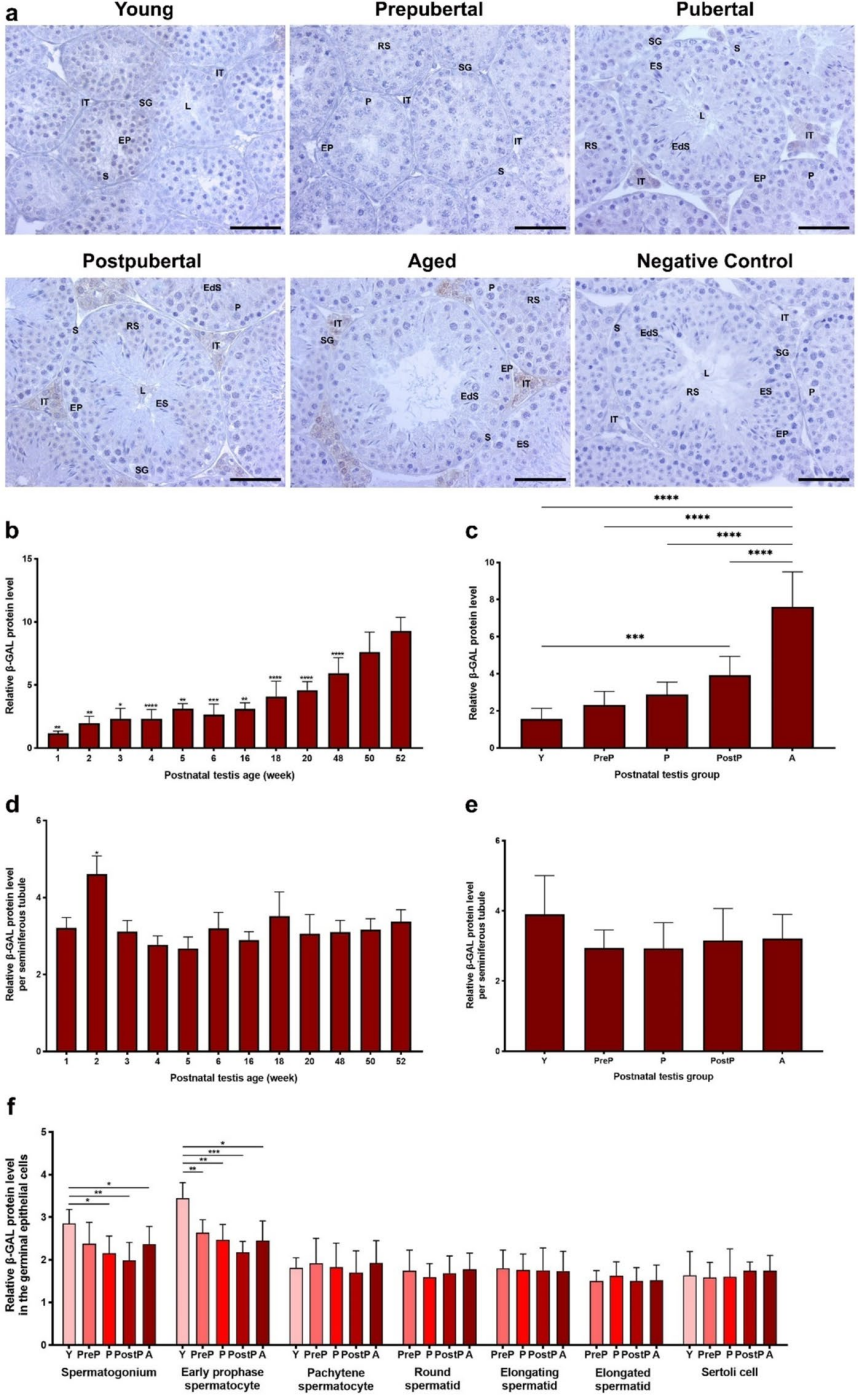
The cellular distribution and relative level of the β-GAL protein in the postnatal mouse testes from early to older ages. **a** Representative micrographs of β-GAL immunostaining in the young (n = 10), prepubertal (n = 8), pubertal (n = 8), postpubertal (n = 12), and aged (n = 17) groups. The micrographs were captured at 400 × original magnification. Scale bars are equal to 50 µm. **b** Relative β-GAL levels in the testes at different ages from 1- to 52-week-old and **c** postnatal testes from the young to the aged groups. **d** Relative β-GAL levels per seminiferous tubule in each age and **e** each group. **f** Relative β-GAL levels in germinal epithelial cells of the postnatal testis groups. The data were analyzed using one-way ANOVA followed by Tukey’s post hoc test. *P* < 0.05 was considered statistically significant. We present values as mean ± standard deviation (SD). *P < 0.05, **P < 0.01, ***P < 0.001, ****P < 0.0001. *Y* young, *PreP* prepubertal, *P* pubertal, *PostP* postpubertal, *A* aged, *SG* spermatogonium, *EP* early prophase spermatocyte, *P* pachytene spermatocyte, *RS* round spermatid, *ES* elongating spermatid, *EdS* elongated spermatid, *S* Sertoli cell, *L* lumen, *IT* intertubular area

In the current study, we initially assessed cellular localization and relative levels of the cellular senescence marker, β-GAL protein, in the postnatal mouse testes from 1- to 52-week-old and postnatal groups **(**Fig. 1**)**. The β-GAL expression was observed in a limited number of spermatogenic cell types such as spermatogonia and pachytene spermatocytes, while an intense staining was evident in the intertubular cells, including Leydig cells **(**Fig. 1a**)**. When we analyzed global β-GAL protein levels in the postnatal testes **(**Fig. 1b**)**, it increased from 1- to 5-week-old (*P* < 0.05), slightly decreased at 6-week and then progressively increased from 16- to 52-week-old (*P* < 0.01). In the postnatal groups **(**Fig. 1c**)**, it increased from the young to the aged groups (*P* < 0.001). As expected, expression of β-GAL, a marker of cellular senescence, reached high levels in the aged mouse testes (*P* < 0.0001). Although we found the highest β-GAL expression in the seminiferous tubules of 2-week-old compared to the other ages (Fig. 1d, *P* < 0.05), there was no significant change between the groups **(**Fig. 1e**)**.

The relative β-GAL level in spermatogonia was higher in the young group compared with the pubertal, postpubertal, and aged groups (Fig. 1f, *P* < 0.05). Parallel to that of spermatogonia, β-GAL was at high level in early prophase spermatocytes in the young group compared with the remaining groups (Fig. 1f, *P* < 0.05). However, no predominant changes were noted in pachytene spermatocytes, round, elongating, and elongated spermatids as well as in Sertoli cells **(**Fig. 1f).

## Expression of KMTs in postnatal mouse testes

### SETD1B protein expression

**Fig. 2:**
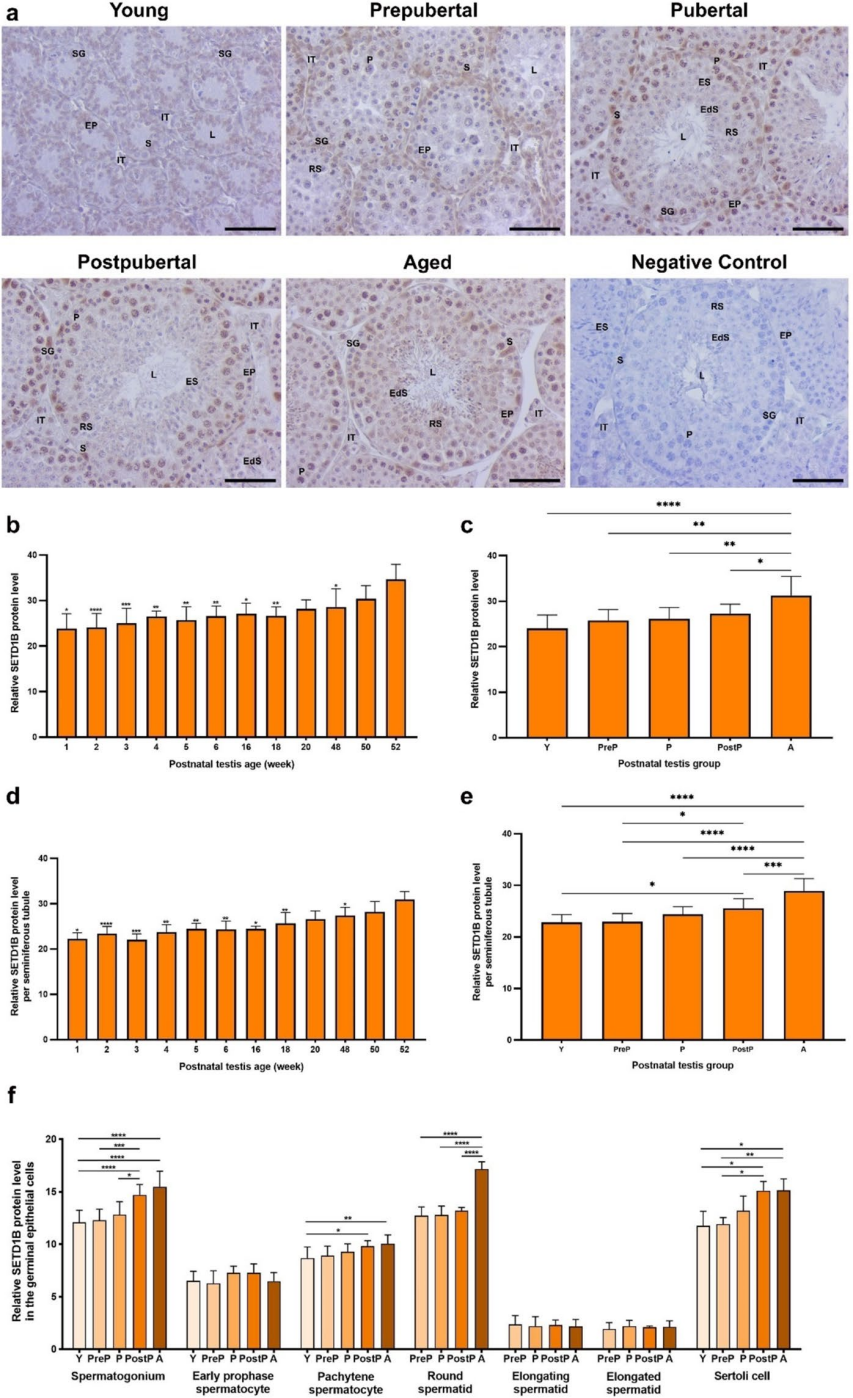
The cellular distribution and relative level of the SETD1B protein in the postnatal mouse testes. **a** Representative micrographs of SETD1B immunostaining in the young (n = 10), prepubertal (n = 8), pubertal (n = 8), postpubertal (n = 12), and aged (n = 17) groups. The micrographs were captured at 400 × original magnification. Scale bars are equal to 50 µm. **b** Relative SETD1B levels in the testes at different ages from 1- to 52-week-old and **c** postnatal testes from the young to the aged groups. **d** Relative SETD1B levels per seminiferous tubule in each age and **e** each group. **f** Relative SETD1B levels in germinal epithelial cells of the postnatal testis groups. The data were analyzed using one-way ANOVA followed by Tukey’s post hoc test. *P* < 0.05 was considered statistically significant. We present values as mean ± standard deviation (SD). *P < 0.05, **P < 0.01, ***P < 0.001, ****P < 0.0001. *Y* young, *PreP* prepubertal, *P* pubertal, *PostP* postpubertal, *A* aged, *SG* spermatogonium, *EP* early prophase spermatocyte, *P* pachytene spermatocyte, *RS* round spermatid, *ES* elongating spermatid, *EdS* elongated spermatid, *S* Sertoli cell, *L* lumen, *IT* intertubular area

The SETD1B protein, which mediates formation of H3K4me3 mark, was expressed in the intertubular area and seminiferous tubules at all ages **(**Fig. 2a**)**. In the seminiferous tubules, there was strong expression in the nuclei of pachytene spermatocytes and Sertoli cells, but weak expression in the remaining spermatogenic cells. In the intertubular area, robust SETD1B expression was observed in nucleus of Leydig cells. When evaluating relative levels of SETD1B protein in the total area **(**Fig. 2b**)**, there was a progressive increase from 1- to 52-week-old testes (*P* < 0.05), except for a slight increase in 4-week and decrease in 18-week. In the postnatal groups, a significant elevation was noted from the young to the aged groups (Fig. 2c, *P* < 0.05). In the seminiferous tubules, SETD1B increased from 1- to 52-week-old testes, except for a minimal reduction in 3-week (Fig. 2d, *P* < 0.05), and similarly increased from the young to the aged groups (Fig. 2e, *P* < 0.05). In each cell type analysis, SETD1B expression demonstrated a gradual increase in spermatogonia (*P* < 0.05), pachytene spermatocytes (*P* < 0.05), round spermatids (*P* < 0.0001), and Sertoli cells from the young to the aged groups (Fig. 2f, *P* < 0.05). However, no discernible change was observed in early prophase spermatocytes, elongating and elongated spermatids between the groups.

### CFP1 protein expression

**Fig. 3:**
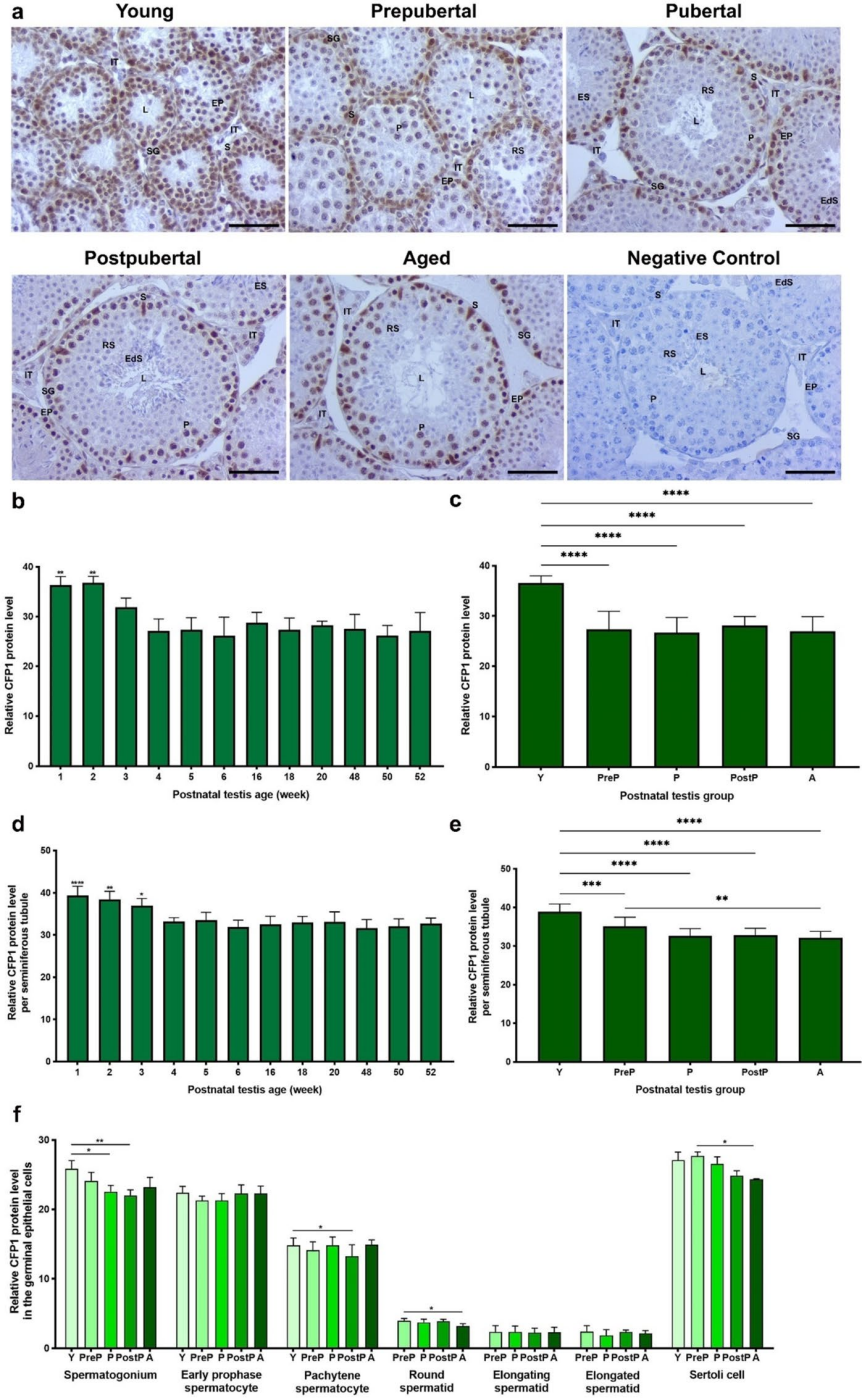
The cellular distribution and relative level of the CFP1 protein in the postnatal mouse testes. **a** Representative micrographs of CFP1 immunostaining in the young (n = 10), prepubertal (n = 8), pubertal (n = 8), postpubertal (n = 12), and aged (n = 17) groups. The micrographs were captured at 400 × original magnification. Scale bars are equal to 50 µm. **b** Relative CFP1 levels in the testes at different ages from 1- to 52-week-old and **c** postnatal testes from the young to the aged groups. **d** Relative CFP1 levels per seminiferous tubule in each age and **e** each group. **f **Relative CFP1 levels in germinal epithelial cells of the postnatal testis groups. The data were analyzed using one-way ANOVA followed by Tukey’s post hoc test. *P* < 0.05 was considered statistically significant. We present values as mean ± standard deviation (SD). *P < 0.05, **P < 0.01, ***P < 0.001, ****P < 0.0001. *Y* young, *PreP* prepubertal, *P* pubertal, *PostP* postpubertal, *A* aged, *SG* spermatogonium, *EP* early prophase spermatocyte, *P* pachytene spermatocyte, *RS* round spermatid, *ES* elongating spermatid, *EdS* elongated spermatid, *S* Sertoli cell, *L* lumen, *IT* intertubular area

The CFP1 protein was observed to be intensely expressed in the nuclei of spermatogonia, early prophase and pachytene spermatocytes, and Sertoli cells of seminiferous tubules of the postnatal testes **(**Fig. 3a**)**. However, the remaining spermatogenic cells, including round, elongating and elongated spermatids, as well as intertubular cells such as Leydig cells, exhibited weak immunostaining. When we evaluated the relative levels of CFP1 protein in the total area, it was at higher levels in 1- and 2-week-old in comparison to the remaining weeks (Fig. 3b, *P* < 0.01). In the postnatal groups, the young group exhibited a higher expression level compared to the remaining groups (Fig. 3c, *P* < 0.0001). The seminiferous tubules of the testes from 1-, 2-, and 3-week-old mice exhibited significantly elevated levels of CFP1 in comparison to the other weeks (Fig. 3d, *P* < 0.05). As anticipated, seminiferous tubules of the young group had a markedly increased expression relative to the remaining groups (Fig. 3e, *P* < 0.001). Furthermore, the prepubertal group demonstrated a higher level than that of the aged group (*P* < 0.01).

Despite absence of noteworthy alterations in early prophase spermatocytes, elongating and elongated spermatids across the groups **(**Fig. 3f**)**, the young group exhibited elevated CFP1 levels in comparison to the pubertal and postpubertal groups in spermatogonia (*P* < 0.05), and compared to the postpubertal group in pachytene spermatocytes (*P* < 0.05). The prepubertal group possessed higher expression levels in both round spermatids and Sertoli cells when compared to the aged group (*P* < 0.05).

### G9A protein expression

**Fig. 4:**
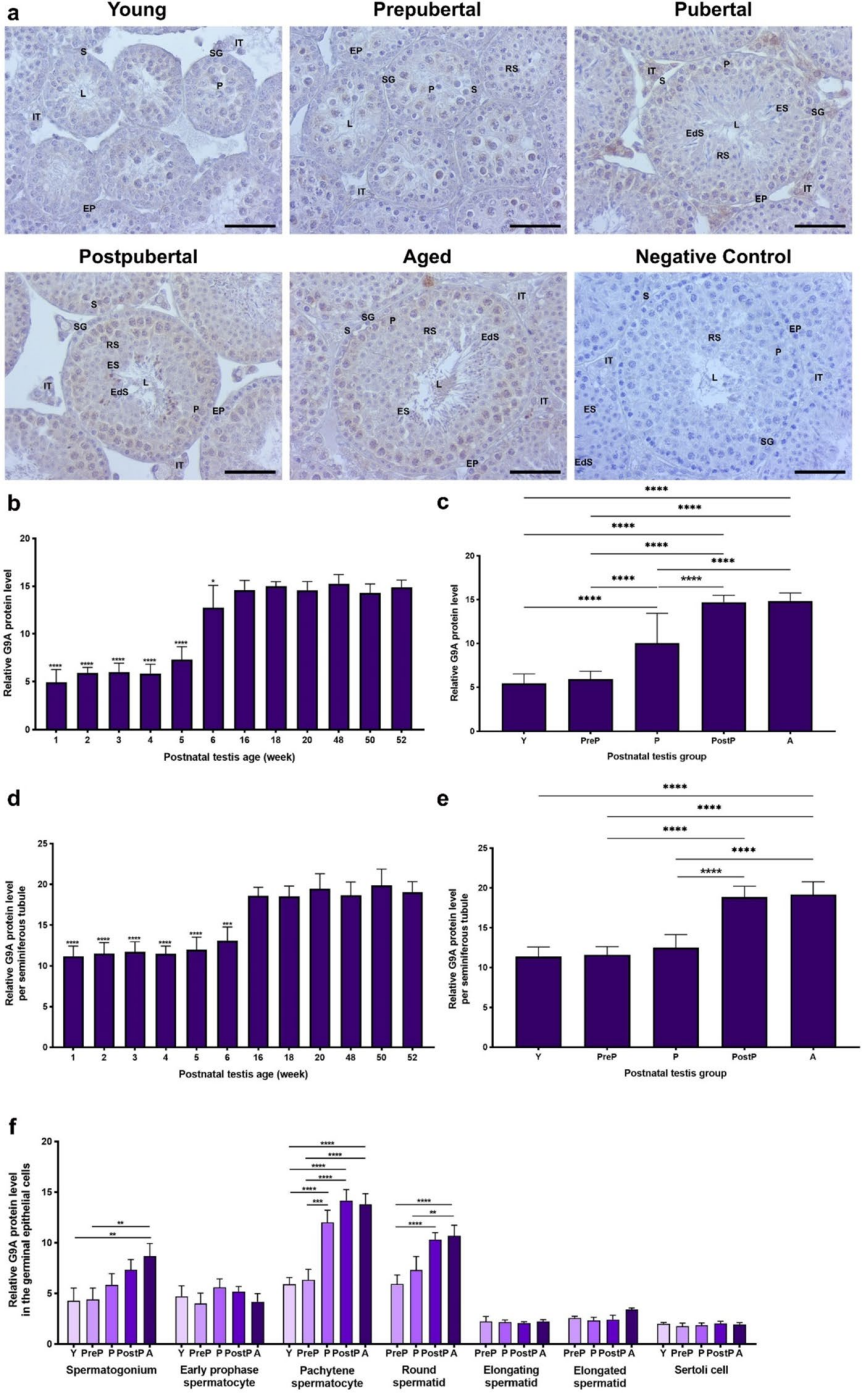
The cellular distribution and relative level of the G9A protein in the postnatal mouse testes. **a** Representative micrographs of G9A immunostaining in the young (n = 10), prepubertal (n = 8), pubertal (n = 8), postpubertal (n = 12), and aged (n = 17) groups. The micrographs were captured at 400 × original magnification. Scale bars are equal to 50 µm. **b** Relative G9A levels in the testes from 1- to 52-week-old and **c** postnatal testes from the young to the aged groups. **d** Relative G9A levels per seminiferous tubule in each age and **e** each group. **f** G9A levels in germinal epithelial cells of the postnatal testis groups. The data were analyzed using one-way ANOVA followed by Tukey’s post hoc test. *P* < 0.05 was considered statistically significant. We present values as mean ± standard deviation (SD). *P < 0.05, **P < 0.01, ***P < 0.001, ****P < 0.0001. *Y* young, *PreP* prepubertal, *P* pubertal, *PostP* postpubertal, *A* aged, *SG* spermatogonium, *EP* early prophase spermatocyte, *P* pachytene spermatocyte, *RS* round spermatid, *ES* elongating spermatid, *EdS* elongated spermatid, *S* Sertoli cell, *L* lumen, *IT* intertubular area

The G9A protein, which is responsible for creating H3K9me2 mark, demonstrated immunoreactivity in intertubular area and seminiferous tubules of the postnatal testes **(**Fig. 4a**)**. An intense expression was observed in pachytene spermatocytes within seminiferous tubules, whereas weak expression was noted in the remaining spermatogenic cells and Sertoli cells. Additionally, the intertubular cells, including Leydig cells, exhibited minimal expression **(**Fig. 4a**)**. The relative G9A expression in the total area was found to be at low levels in 1-, 2-, 3-, 4-, and 5-week-old (*P* < 0.0001), and then reached high levels in 6–52-week-old (Fig. 4b, *P* < 0.05). A progressive increase in G9A level was observed among the groups, with the young group exhibiting the lowest level and the postpubertal and aged groups exhibiting highest level (Fig. 4c, *P* < 0.0001).

In seminiferous tubules, the 16–52-week ages had higher expression compared to the 1–6-week ages (Fig. 4d, *P* < 0.001). The G9A expression in the postpubertal and aged groups showed higher levels than that of the young, prepubertal, and pubertal groups (Fig. 4e, *P* < 0.0001). Despite absence of notable discrepancies in early prophase spermatocytes, elongating and elongated spermatids, and Sertoli cells across the groups, the G9A levels in spermatogonia, pachytene spermatocytes, and round spermatids exhibited an increase from the young to the postpubertal/aged groups (Fig. 4f, *P* < 0.01).

### SETDB1 protein expression

**Fig. 5:**
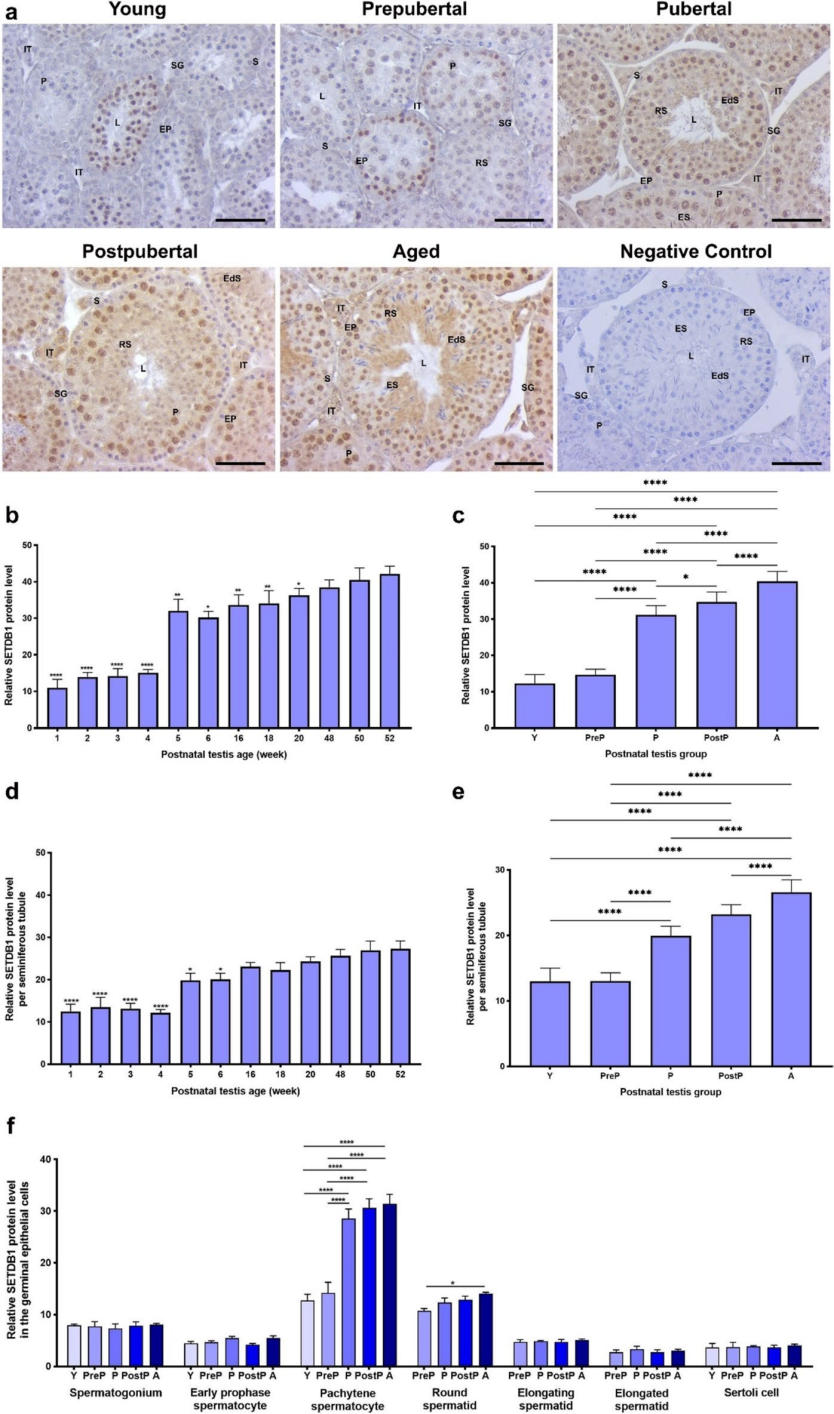
The cellular distribution and relative level of the SETDB1 protein in the postnatal mouse testes. **a** Representative micrographs of SETDB1 immunostaining in the young (n = 10), prepubertal (n = 8), pubertal (n = 8), postpubertal (n = 12), and aged (n = 17) groups. The micrographs were captured at 400 × original magnification. Scale bars are equal to 50 µm. **b** SETDB1 levels in the testes from 1- to 52-week-old ages and **c** postnatal testes from the young to the aged groups. **d** SETDB1 levels per seminiferous tubule in each age and **e.** each group. **f** SETDB1 levels in germinal epithelial cells of the postnatal testis groups. The data were analyzed using one-way ANOVA followed by Tukey’s post hoc test. *P* < 0.05 was considered statistically significant. We present values as mean ± standard deviation (SD). *P < 0.05, **P < 0.01, ***P < 0.001, ****P < 0.0001. *Y* young, *PreP* prepubertal, *P* pubertal; *PostP* postpubertal, *A* aged, *SG* spermatogonium, *EP* early prophase spermatocyte, *P* pachytene spermatocyte, *RS* round spermatid, *ES* elongating spermatid, *EdS* elongated spermatid, *S* Sertoli cell, *L* lumen, *IT* intertubular area

The cellular distribution and relative levels of another KMT, SETDB1 (a H3K9me3 methyltransferase), were also analyzed in the postnatal testes. The results indicated that it was intensely localized in pachytene spermatocytes, round and elongating spermatids **(**Fig. 5a**)**. In addition to weak expression in Sertoli cells, SETDB1 was observed in the nucleus and cytoplasm of intertubular cells, including Leydig cells. The relative SETDB1 expression in total area of the postnatal testes **(**Fig. 5b**)** exhibited low levels at 1-, 2-, 3-, and 4-week-old (*P* < 0.0001), and then demonstrated a gradual increase from 5- to 52-week-old (*P* < 0.05), with the exception of a slight decline observed at 6-week-old. The SETDB1 expression increased in a progressive manner from the young to the aged groups (Fig. 5c, *P* < 0.05). It was at low levels in the seminiferous tubules of 1-, 2-, 3-, and 4-week (*P* < 0.0001) and subsequently increased from 5- to 52-week-old except for a slight decrease in 18-week-old (Fig. 5d, *P *< 0.05).

The level of SETDB1 in seminiferous tubules of the postnatal groups demonstrated an increase from the young/prepubertal to the aged groups (Fig. 5e, *P* < 0.0001). Despite absence of alterations in spermatogonia, early prophase spermatocytes, elongating and elongated spermatids, and Sertoli cells, the pachytene spermatocytes (*P* < 0.0001) and round spermatids (*P* < 0.05) showed a gradual increase from the young to the aged groups (Fig. 5f).

### SETD2 protein expression

**Fig. 6:**
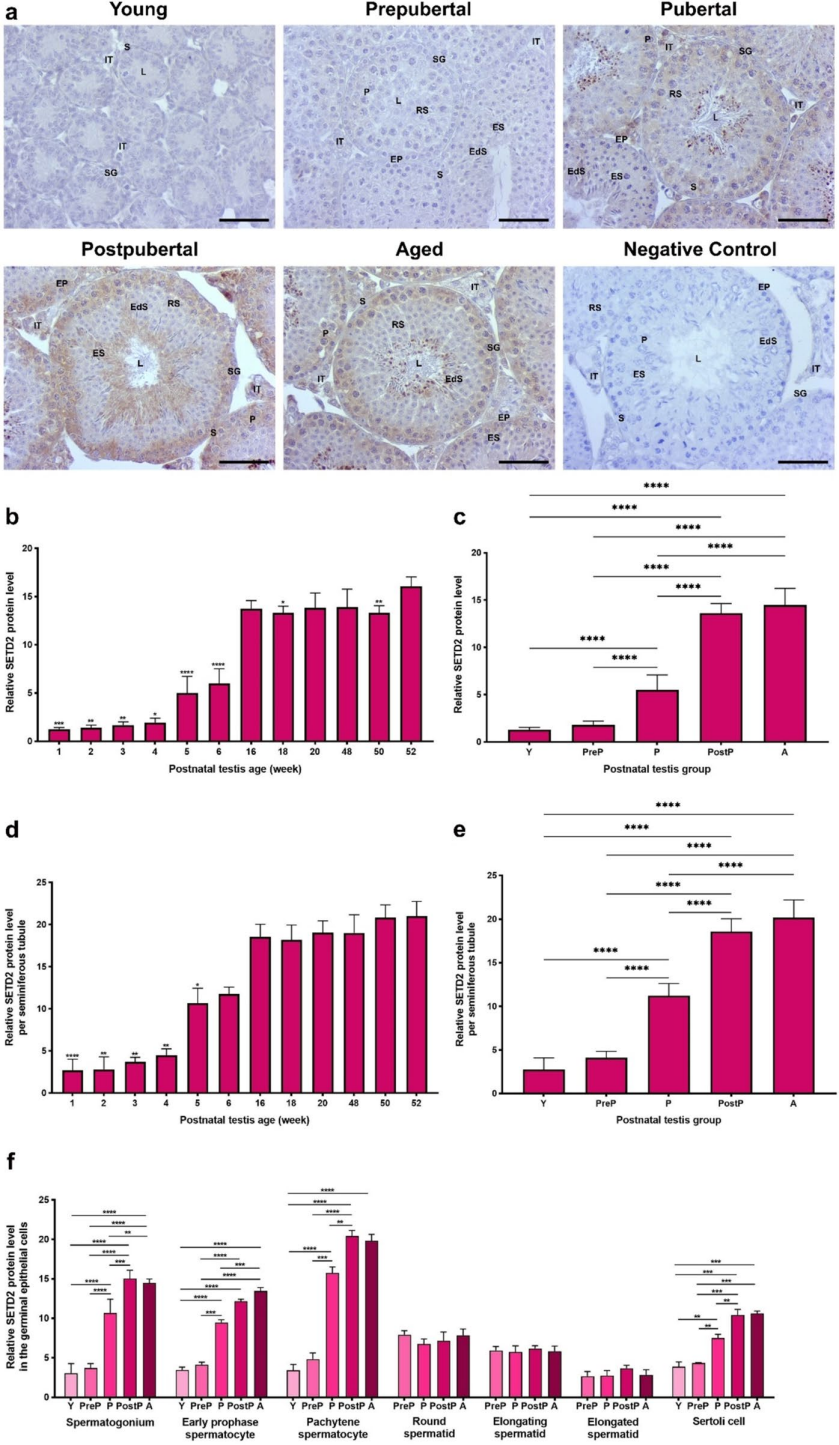
The cellular distribution and relative level of the SETD2 protein in the postnatal mouse testes. **a** Representative micrographs of SETD2 immunostaining in the young (n = 10), prepubertal (n = 8), pubertal (n = 8), postpubertal (n = 12), and aged (n = 17) groups. The micrographs were captured at 400 × original magnification. Scale bars are equal to 50 µm. **b** SETD2 levels in the testes from 1- to 52-week-old ages and **c** postnatal testes from the young to the aged groups. **d** SETD2 levels per seminiferous tubule in each age and **e** each group. **f** SETD2 levels in germinal epithelial cells of the postnatal testis groups. The data were analyzed using one-way ANOVA followed by Tukey’s post hoc test. *P* < 0.05 was considered statistically significant. We present values as mean ± standard deviation (SD). *P < 0.05, **P < 0.01, ***P < 0.001, ****P < 0.0001. *Y* young, *PreP* prepubertal, *P* pubertal, *PostP* postpubertal, *A* aged, *SG* spermatogonium, *EP* early prophase spermatocyte, *P* pachytene spermatocyte, *RS* round spermatid, *ES* elongating spermatid, *EdS* elongated spermatid, *S* Sertoli cell, *L* lumen, *IT* intertubular area

The SETD2 protein was highly expressed in early prophase and pachytene spermatocytes, whereas the remaining germinal epithelial cells displayed minimal expression **(**Fig. 6a**)**. The intertubular cells such as Leydig cells had low expression. In the total (Fig. 6b) and seminiferous tubule area (Fig. 6d) analyses, low expression was observed in the early weeks (from 1- to 4-week old, *P* < 0.05), which increased in 5- and 6-week ages (*P* < 0.05) and reached high levels in the ages from 16- to 52-week old (*P* < 0.05). In the postnatal groups, relative SETD2 levels elevated from the young to the aged groups (Fig. 6c, e, *P* < 0.0001). Upon evaluating SETD2 expression in the spermatogenic cells **(**Fig. 6f**)**, no discernible difference was observed in round, elongating and elongated spermatids. However, a notable enhancement was noted in early prophase spermatocytes (*P* < 0.001) and Sertoli cells (*P* < 0.01) from the young to the aged groups. In spermatogonia (*P* < 0.01) and pachytene spermatocytes (*P* < 0.01), SETD2 expression increased from the young to the postpubertal/aged groups.

## Expression of the histone methylation marks in the postnatal mouse testes

### H3K4me3 expression

**Fig. 7:**
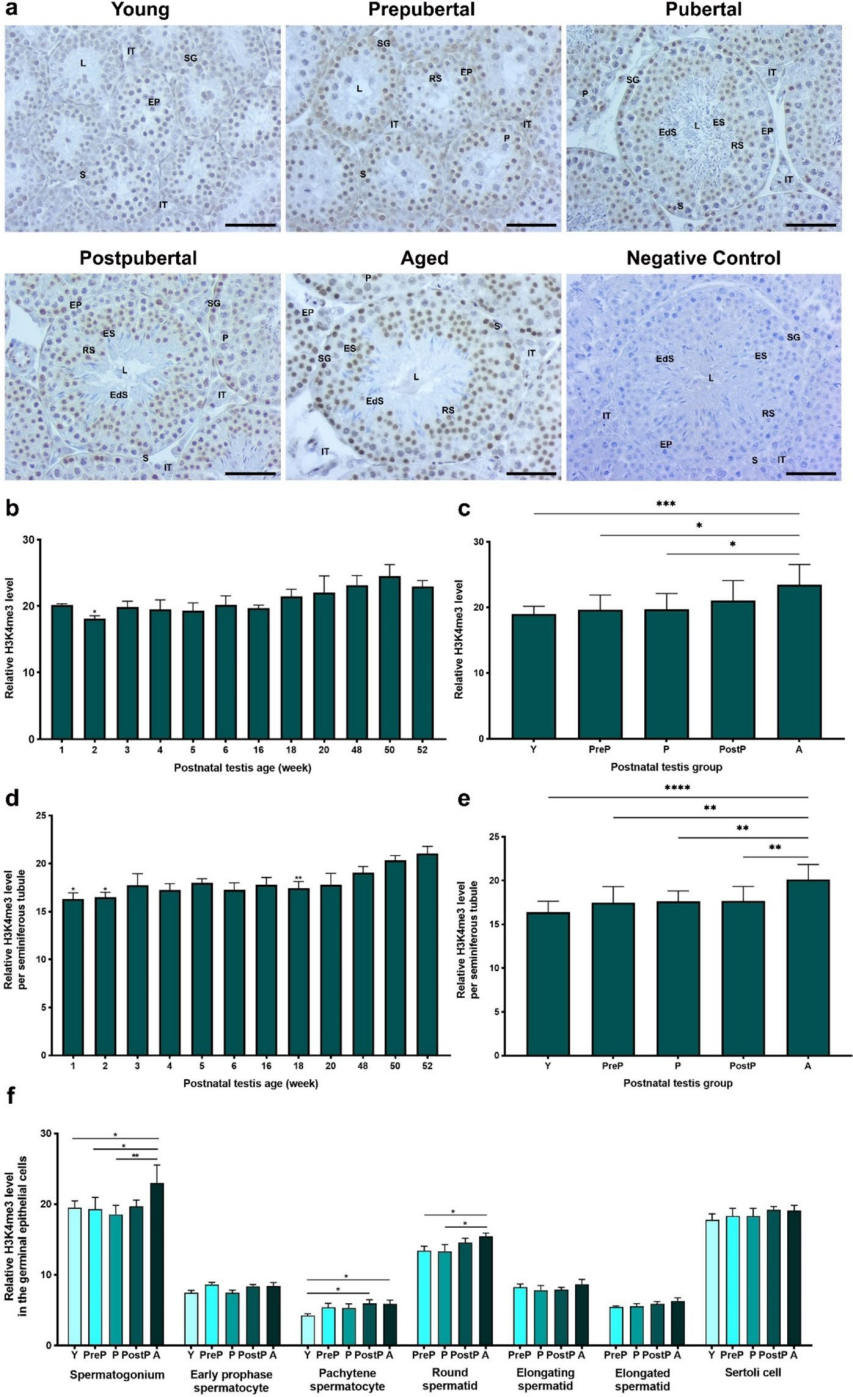
The cellular distribution and relative level of H3K4me3 in the postnatal mouse testes. **a** Representative micrographs of H3K4me3 immunostaining in the young (n = 10), prepubertal (n = 8), pubertal (n = 8), postpubertal (n = 12), and aged (n = 17) groups. The micrographs were captured at 400 × original magnification. Scale bars are equal to 50 µm. **b** Relative H3K4me3 levels in the testes from 1- to 52-week-old ages and **c** postnatal testes from the young to the aged groups. **d** Relative H3K4me3 levels per seminiferous tubule in each age and **e** each group. **f** Relative H3K4me3 levels in germinal epithelial cells of the postnatal testis groups. The data were analyzed using one-way ANOVA followed by Tukey’s post hoc test. *P* < 0.05 was considered statistically significant. We present values as mean ± standard deviation (SD). *P < 0.05, **P < 0.01, ***P < 0.001, ****P < 0.0001. *Y* young, *PreP* prepubertal, *P* pubertal, *PostP* postpubertal, *A* aged, *SG* spermatogonium, *EP* early prophase spermatocyte, *P* pachytene spermatocyte, *RS* round spermatid, *ES* elongating spermatid, *EdS* elongated spermatid, *S* Sertoli cell, *L* lumen, *IT* intertubular area

H3K4me3 is highly expressed in the nuclei of spermatogonial cells and round spermatids **(**Fig. 7a**)**. However, there was very weak expression in early and pachytene spermatocytes, elongating and elongated spermatids, Sertoli cells as well as in the intertubular cells such as Leydig cells. A total analysis revealed that H3K4me3 level was significantly lower in the testes of 2-week-old when compared to those of the remaining weeks (Fig. 7b, *P* < 0.05). In the postnatal groups, it reached the highest level in the aged group in comparison to the remaining groups (Fig. 7c, *P* < 0.05). Upon evaluation of the seminiferous tubules, it was observed that the relative H3K4me3 levels were low in the testes of 1-, 2-, and 18-week-old, but exhibited a marked increase in 48-, 50-, and 52-week (Fig. 7d, *P* < 0.05). In the postnatal groups, H3K4me3 showed the lowest level in the young group and reached its highest level in the aged group (Fig. 7e, *P* < 0.01). Despite absence of discernible differences in early prophase spermatocytes, elongating and elongated spermatids, and Sertoli cells, the aged/postpubertal group exhibited increased levels of H3K4me3 in spermatogonia, pachytene spermatocytes, and round spermatids compared to the other groups (Fig. 7f, *P* < 0.05).

### H3K9me2 expression

**Fig. 8:**
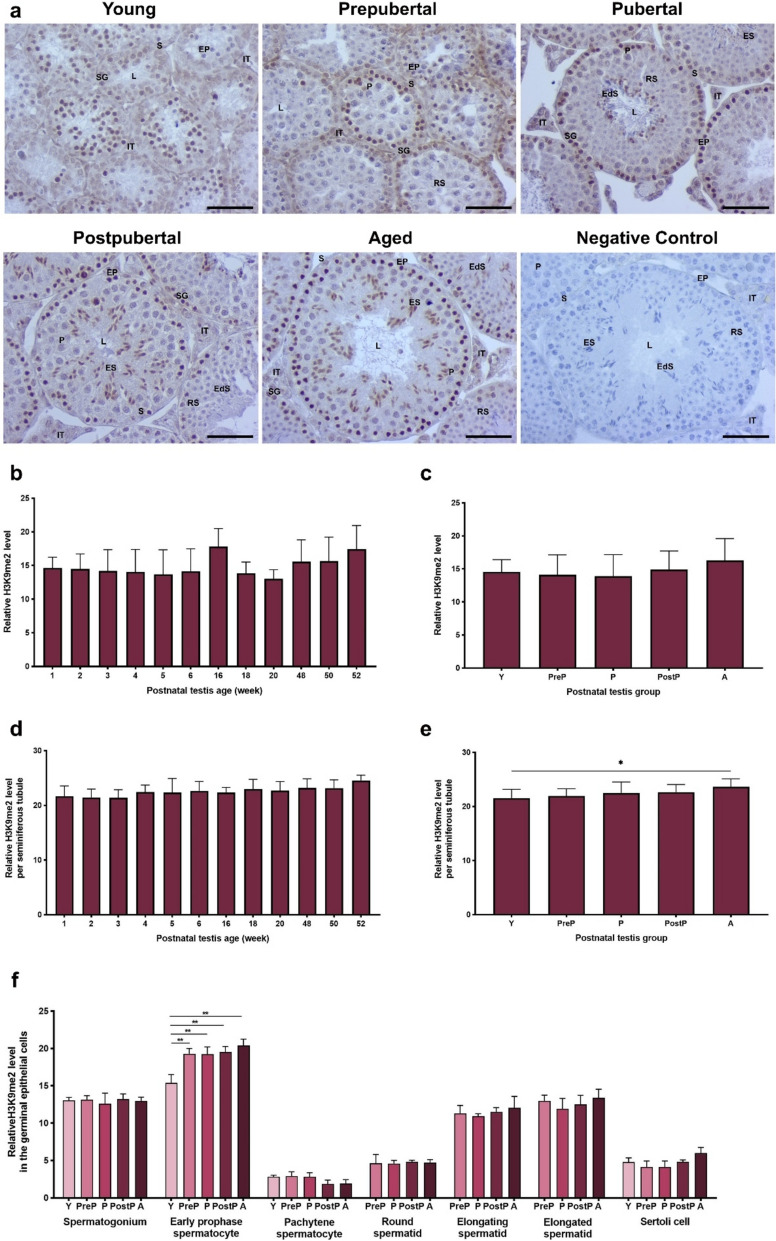
The cellular distribution and relative level of H3K9me2 in the postnatal mouse testes. **a** Representative micrographs of H3K9me2 immunostaining in the young (n = 10), prepubertal (n = 8), pubertal (n = 8), postpubertal (n = 12), and aged (n = 17) groups. The micrographs were captured at 400 × original magnification. Scale bars are equal to 50 µm. **b** Relative H3K9me2 levels in the testes from 1- to 52-week-old ages and **c** postnatal testes from the young to the aged groups. **d** H3K9me2 levels per seminiferous tubule in each age and **e** each group. **f** H3K9me2 levels in germinal epithelial cells of the postnatal testis groups. The data were analyzed using one-way ANOVA followed by Tukey’s post hoc test. *P* < 0.05 was considered statistically significant. We present values as mean ± standard deviation (SD). *P < 0.05, **P < 0.01, ***P < 0.001, ****P < 0.0001. *Y* young, *PreP* prepubertal, *P* pubertal, *PostP* postpubertal, *A* aged, *SG* spermatogonium, *EP* early prophase spermatocyte, *P* pachytene spermatocyte, *RS* round spermatid, *ES* elongating spermatid, *EdS* elongated spermatid, *S* Sertoli cell, *L* lumen, *IT* intertubular area

The H3K9me2 mark was strongly accumulated in the nuclei of spermatogonia, early prophase spermatocytes, and elongating spermatids **(**Fig. 8a**)**. However, it showed very low accumulation in pachytene spermatocytes, round and elongated spermatids as well as in Sertoli cells. The total analysis revealed no statistically significant alterations across the weeks **(**Fig. 8b**)** and postnatal groups **(**Fig. 8c**)**. Although no difference was observed in seminiferous tubules of the weeks **(**Fig. 8d**)**, the aged group exhibited a higher level of H3K9me2 when compared to the young group **(**Fig. 8e, *P* < 0.05). The level of H3K9me2 in early prophase spermatocytes exhibited the lowest immunostaining in the young group compared to the other groups (*P* < 0.01), whereas no differences were observed in the remaining spermatogenic and Sertoli cells **(**Fig. 8f**)**.

### H3K9me3 expression

**Fig. 9:**
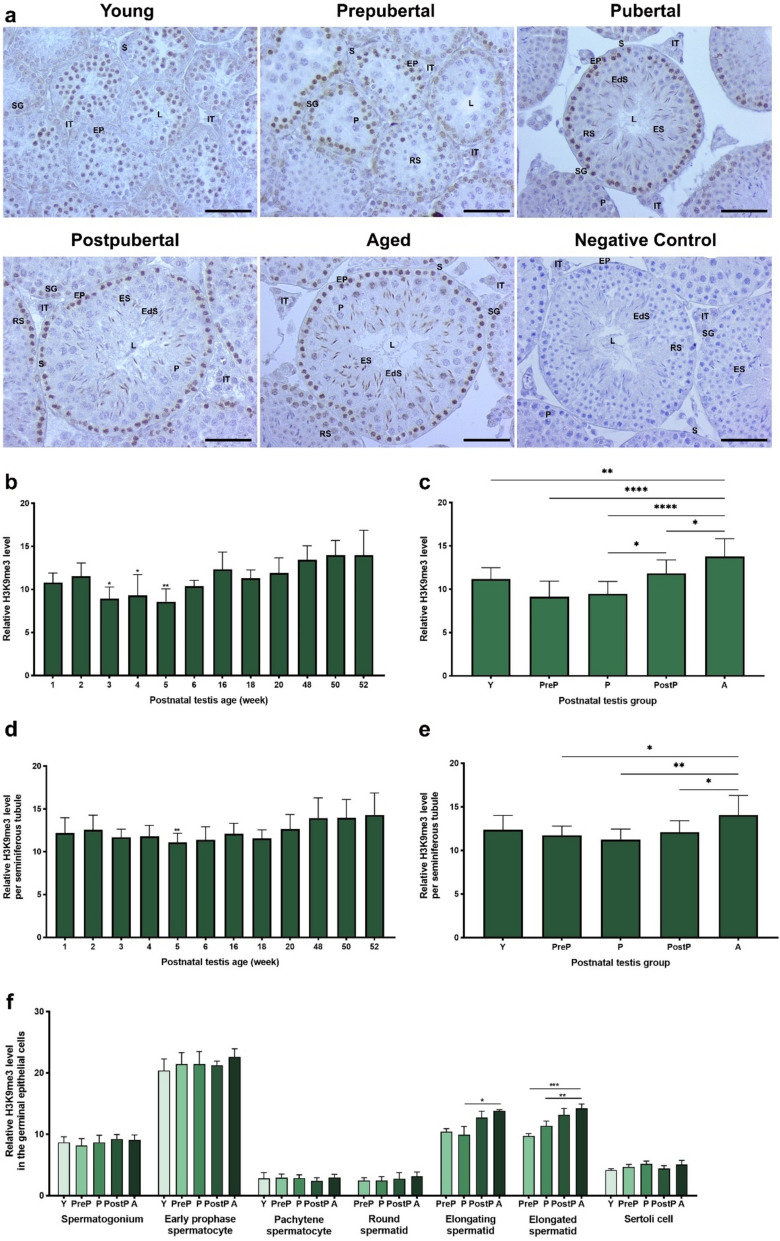
The cellular distribution and relative level of H3K9me3 in the postnatal mouse testes. **a** Representative micrographs of H3K9me3 immunostaining in the young (n = 10), prepubertal (n = 8), pubertal (n = 8), postpubertal (n = 12), and aged (n = 17) groups. The micrographs were captured at 400 × original magnification. Scale bars are equal to 50 µm. **b** Relative H3K9me3 levels in the testes from 1- to 52-week-old ages and **c** postnatal testes from the young to the aged groups. **d** H3K9me3 levels per seminiferous tubule in each age and **e** each group. **f** H3K9me3 levels in germinal epithelial cells of the postnatal testis groups. The data were analyzed using one-way ANOVA followed by Tukey’s post hoc test. *P* < 0.05 was considered statistically significant. We present values as mean ± standard deviation (SD). *P < 0.05; **P < 0.01; ***P < 0.001; ****P < 0.0001. *Y* young, *PreP* prepubertal, *P* pubertal, *PostP* postpubertal, *A* aged, *SG* spermatogonium, *EP* early prophase spermatocyte, *P* pachytene spermatocyte, *RS* round spermatid, *ES* elongating spermatid, *EdS* elongated spermatid, *S* Sertoli cell, *L* lumen, *IT* intertubular area

Similar to the H3K9me2 expression, the H3K9me3 mark was expressed in the nuclei of spermatogonia, early prophase spermatocytes, and elongating spermatids. However, there was weak expression in pachytene spermatocytes, round spermatids, elongated spermatids, and Sertoli cells **(**Fig. 9a**)**. The total analysis revealed a notable decline in expression levels in the testes of 3-, 4-, and 5-week-old (*P* < 0.05), which subsequently exhibited elevated levels in the aged testes, specifically those of 48-, 50-, and 52-week-old **(**Fig. 9b**)**. In the postnatal groups, H3K9me3 levels decreased from the young to the prepubertal groups, which then gradually increased toward the aged group **(**Fig. 9c,* P* < 0.05). Upon evaluating the expression per seminiferous tubules, it was observed that there were notable changes in expression across the weeks, with the lowest level observed in 5-week-old (*P* < 0.01) and highest levels observed in 48-, 50-, and 52-week-old **(**Fig. 9d**)**. In seminiferous tubules of the postnatal groups, there was a decline in the relative H3K9me3 levels from the young to the pubertal groups, which subsequently increased toward the aged group **(**Fig. 9e, *P* < 0.05). Despite absence of notable discrepancies in spermatogonia, early prophase and pachytene spermatocytes, round spermatids, and Sertoli cells, the elongating (*P* < 0.05) and elongated (*P* < 0.01) spermatids exhibited a gradual elevation in H3K9me3 levels from the young to the aged groups, with the exception of a minimal decline in elongating spermatids of the pubertal group **(**Fig. 9f**)**.

### H3K36me3 expression

**Fig. 10:**
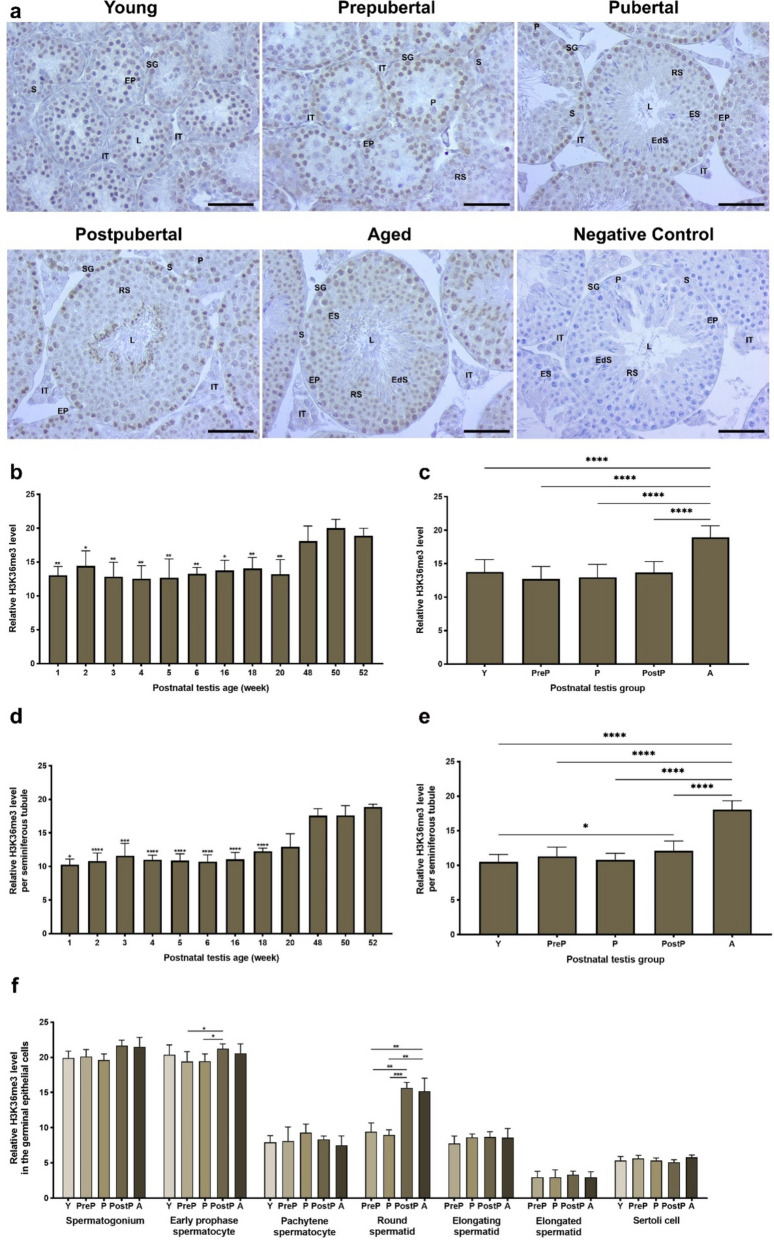
The cellular distribution and relative level of H3K36me3 in the postnatal mouse testes. **a** Representative micrographs of H3K36me3 immunostaining in the young (n = 10), prepubertal (n = 8), pubertal (n = 8), postpubertal (n = 12), and aged (n = 17) groups. The micrographs were captured at 400 × original magnification. Scale bars are equal to 50 µm. **b** Relative H3K36me3 levels in the testes from 1- to 52-week-old ages and **c** postnatal testes from the young to the aged groups. **d** Relative H3K36me3 levels per seminiferous tubule in each age and **e** each group. **f** Relative H3K36me3 levels in germinal epithelial cells of the postnatal testis groups. The data were analyzed using one-way ANOVA followed by Tukey’s post hoc test. *P* < 0.05 was considered statistically significant. We present values as mean ± standard deviation (SD). *P < 0.05, **P < 0.01, ***P < 0.001, ****P < 0.0001. *Y* young, *PreP* prepubertal, *P* pubertal, *PostP* postpubertal, *A* aged, *SG* spermatogonium, *EP* early prophase spermatocyte, *P* pachytene spermatocyte, *RS* round spermatid, *ES* elongating spermatid, *EdS* elongated spermatid, *S* Sertoli cell, *L* lumen, *IT* intertubular area

The immunoexpression of H3K36me3 was markedly evident in spermatogonia and early prophase spermatocytes **(**Fig. 10a**)**. However, it was relatively low in pachytene spermatocytes and round spermatids. A total analysis demonstrated that diminished H3K36me3 levels observed in the testes aged from 1- to 20-week-old exhibited a notable increase in 48-, 50-, and 52-week-old (Fig. 10b, *P* < 0.05). In the postnatal groups, the H3K36me3 level reached its highest level in the aged group (Fig. 10c, *P* < 0.0001). It is noteworthy that seminiferous analysis yielded comparable results with the total analysis among the weeks (Fig. 10d, *P* < 0.05) and postnatal groups (Fig. 10e, *P* < 0.0001). The early prophase spermatocytes of the postpubertal group had higher expression than the prepubertal and pubertal groups (*P* < 0.05), and round spermatids of the postpubertal and aged groups (*P* < 0.01) exhibited elevated levels of H3K36me3 when compared to the remaining groups **(**Fig. 10f**)**. It is important to note that we found no significant differences in spermatogonia, pachytene spermatocytes, elongating spermatids, elongated spermatids, and Sertoli cells between the groups **(**Fig. 10f**)**.

## Expression of pS2 and cCASP3 in the postnatal mouse testes

### pS2 expression

**Fig. 11:**
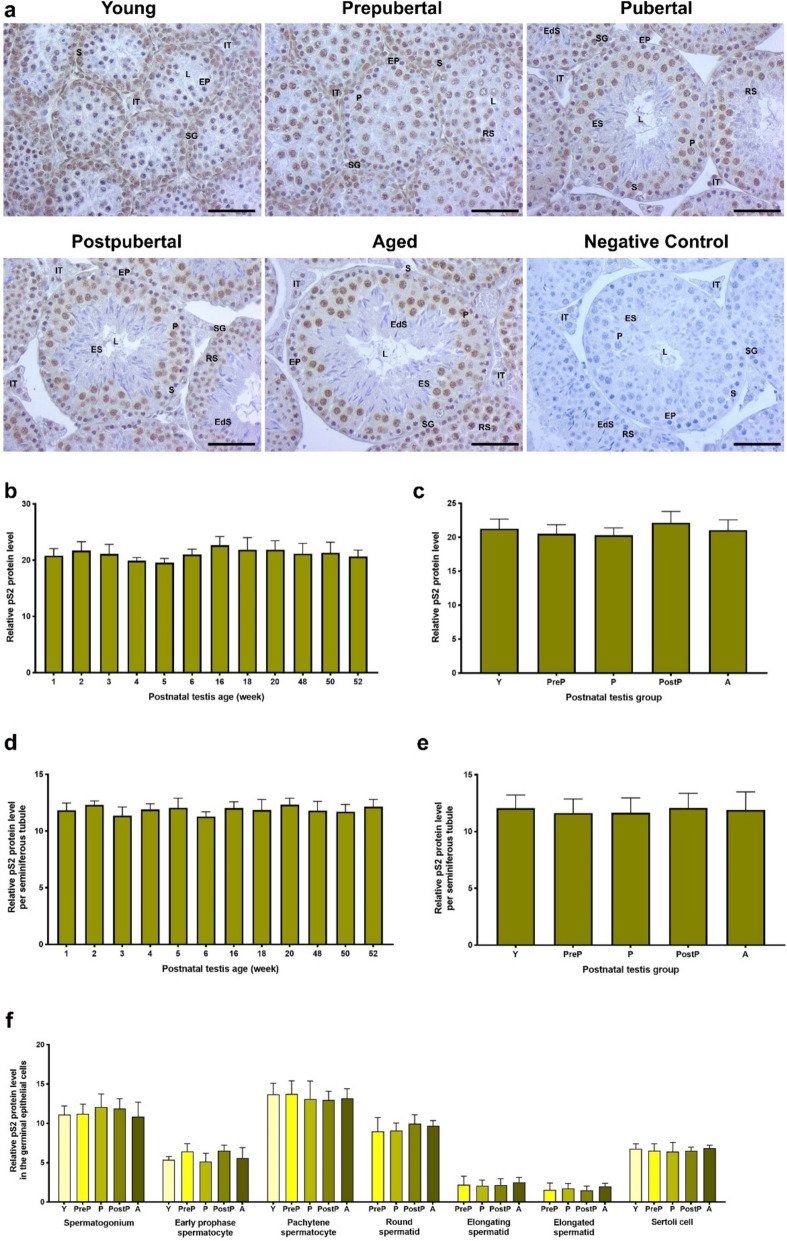
The cellular distribution and relative level of the pS2 protein in the postnatal mouse testes. **a** Representative micrographs of pS2 immunostaining in the young (n = 10), prepubertal (n = 8), pubertal (n = 8), postpubertal (n = 12), and aged (n = 17) groups. The micrographs were captured at 400 × original magnification. Scale bars are equal to 50 µm. **b** pS2 levels in the testes from 1- to 52-week-old ages and **c** postnatal testes from the young to the aged groups. **d** pS2 levels per seminiferous tubule in each age and **e** each group. **f** pS2 levels in germinal epithelial cells of the postnatal testis groups. The data were analyzed using one-way ANOVA followed by Tukey’s post hoc test. *P* < 0.05 was considered statistically significant. We present values as mean ± standard deviation (SD). *P < 0.05, **P < 0.01, ***P < 0.001, ****P < 0.0001. *Y* young, *PreP* prepubertal, *P* pubertal, *PostP* postpubertal, *A* aged, *SG* spermatogonium, *EP* early prophase spermatocyte, *P* pachytene spermatocyte, *RS* round spermatid, *ES* elongating spermatid, *EdS* elongated spermatid, *S* Sertoli cell, *L* lumen, *IT* intertubular area

pS2 demonstrated a pronounced expression within the nuclear regions of pachytene spermatocytes and Sertoli cells **(**Fig. 11a**)**. Conversely, a low expression was observed in the nuclei of spermatogonia and round spermatids, as well as in the intertubular cells, including Leydig cells. In analysis of the total area **(**Fig. 11b, c**)**, seminiferous tubules **(**Fig. 11d, e**)**, and each germinal epithelial cell **(**Fig. 11f**)**, although there were small changes in the pS2 expression among the weeks and groups, no statistically significant differences were identified.

### cCASP3 expression

**Fig. 12:**
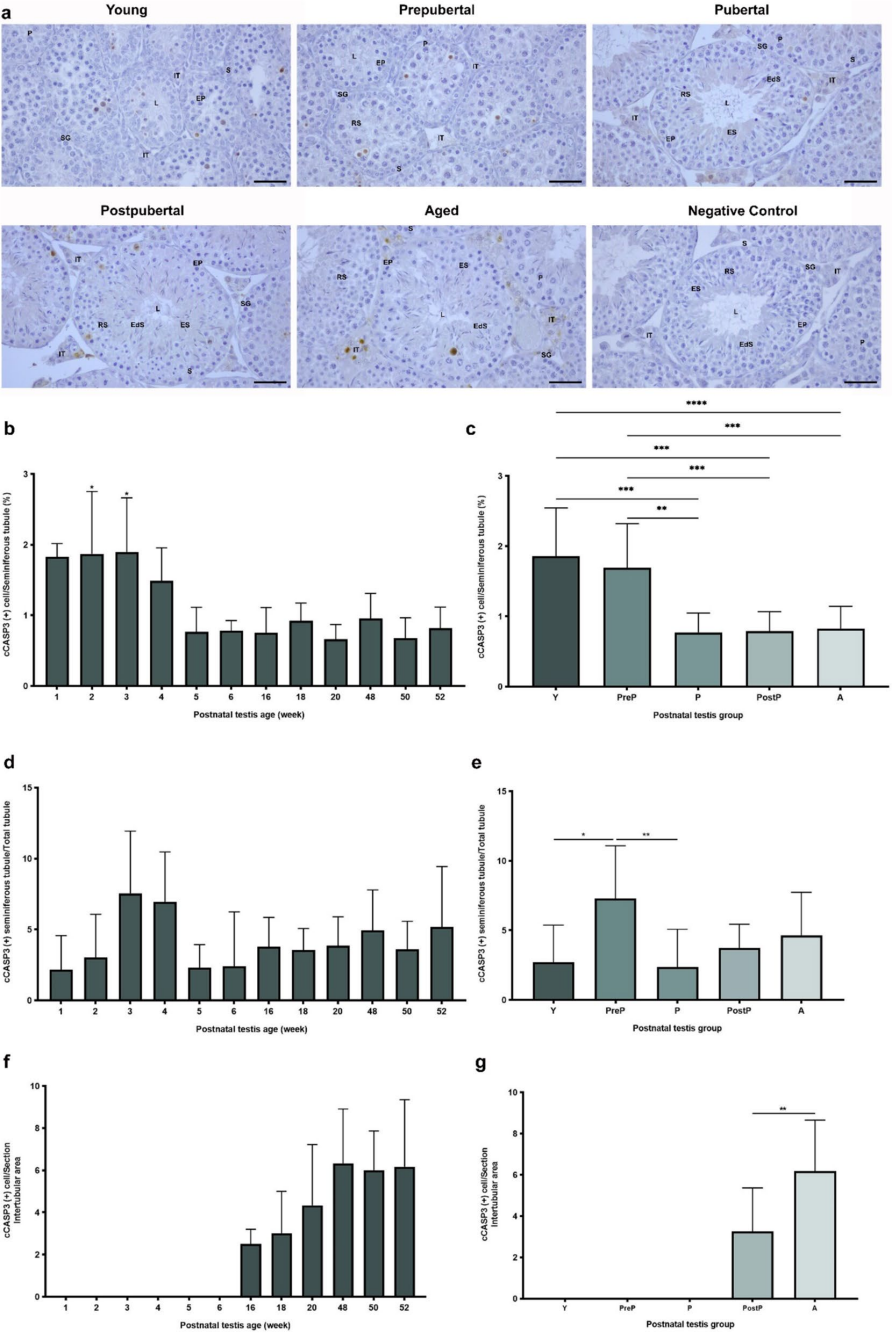
The cellular distribution and relative level of cCASP3 in the postnatal mouse testes. **a** Representative micrographs of cCASP3 immunostaining in the young (n = 10), prepubertal (n = 8), pubertal (n = 8), postpubertal (n = 12), and aged (n = 17) groups. The micrographs were captured at 400 × original magnification. Scale bars are equal to 50 µm. **b** Percentage of cCASP3 positive cells in the testes from 1- to 52-week-old ages and **c** postnatal testes from the young to the aged groups. **d** cCASP3 positive seminiferous tubule in each age and **e** each group. **f** Percentage of cCASP3 positive cells in the intertubular area in each age. **g** Percentage of cCASP3 positive cells in the intertubular area in each postnatal testis group. The data were analyzed using one-way ANOVA followed by Tukey’s post hoc test. *P* < 0.05 was considered statistically significant. We present values as mean ± standard deviation (SD). *P < 0.05, **P < 0.01, ***P < 0.001, ****P < 0.0001. *Y* young, *PreP* prepubertal, *P* pubertal, *PostP* postpubertal, *A* aged, *SG* spermatogonium, *EP* early prophase spermatocyte, *P* pachytene spermatocyte, *RS* round spermatid, *ES* elongating spermatid, *EdS* elongated spermatid, *S* Sertoli cell, *L* lumen, *IT* intertubular area

The expression of cCASP3 was observed in germinal epithelial cells of the seminiferous tubules and in the intertubular cells of postnatal testes **(**Fig. 12a**)**. The percentage of cCASP3 positive cells in seminiferous tubules were evaluated in the weeks, with higher levels observed in 1- to 4-week-old ages, followed by a decline in the remaining weeks **(**Fig. 12b, *P* < 0.05). As expected, the young and prepubertal groups exhibited increased percentage of cCASP3 positive cells relative to the pubertal, postpubertal, and aged groups in the seminiferous tubules **(**Fig. 12c, *P* < 0.01). There were changes in the percentage of cCASP3 positive seminiferous tubules among the total seminiferous tubules in the weeks from 1- to 52-week-old **(**Fig. 12d**)**, and the prepubertal group had higher percentage of cCASP3 positive seminiferous tubules when compared to the young (*P* < 0.05) and pubertal (*P* < 0.01) groups **(**Fig. 12e**)**. The cCASP3-positive intertubular cells was observed in the testes from 16- to 52-week-old **(**Fig. 12f**)**. While there was absence of cCASP3-positive intertubular cells in the young, prepubertal, and pubertal groups, the aged group exhibited a higher percentage in comparison to the postpubertal group **(**Fig. 12g, *P* < 0.01).

## Discussion

The β-GAL expression, which is utilized as a marker of cellular aging (Debacq–Chainiaux et al. 2009), showed an increase accompanying with biological aging. Consistent with our results, a recent study demonstrated a significant increase in β-GAL activity in the testes of 24-month-old mice compared to those of a younger age (2 months old) (Endo et al. 2024). In the current study, we observed a notable accumulation of β-GAL in cytoplasm of the intertubular cells including Leydig cells suggesting that Leydig cells may be more susceptible to age-related alterations possibly due to their testicular localization and decrease in the reproductive hormones such as testosterone.

The absence or changed expression of KMTs and subsequent inability to establish histone methylation marks in the spermatogenic cells may result in meiotic arrest, chromosome missegregation, DNA damage, and aberrant homologous recombination [Reviewed in (Bilmez and Ozturk 2023)]. One of these KMTs, SETD1B, is a basic component of SET1 complex that catalyzes H3K4 methylation (Lee et al. 2007). Thus, transcriptional activity-associated H3K4me3 mark is generated at actively transcribing gene regions (Lee et al. 2007). A review of the literature revealed no studies that have evaluated SETD1B protein expression in mouse testes during biological aging. According to the analyses in the current study, the increase in SETD1B protein level with testicular aging may be related to the need to increase the levels of transcription-associated H3K4me3, since transcriptional activity in spermatogenic cells is known to decrease in some genes accompanying with age (Liao et al. 2021; Fice and Robaire 2023).

CFP1 is a principal component of the SETD1 complex and functions as a DNA-binding factor, facilitating formation of H3K4me3 mark (Shinsky et al. 2015). Expectedly, CFP1 reaches high levels within the promoter and transcription start site of target genes to increase H3K4me3 accumulation and thereby enhance gene expression in pachytene spermatocytes at the first meiotic prophase (Ki et al. 2022). Absence of the *Cxxc1* gene encoding the CFP1 protein (Jiang, Zhang et al. 2020, Ki et al. 2022) led to decreased H3K4me3 levels at PR domain-containing protein 9 (PRDM9) binding sites in leptotene and zygotene spermatocytes as well as impaired DNA double-strand break (DSB) repair, homologous recombination and acrosome formation. These results confirm the critical role of CFP1 for establishment of H3K4 methylation, which is essential for homologous recombination, chromosome segregation, and meiotic progression during spermatogenesis. The elevated CFP1 expression in the young group may be attributed to including greater numbers of spermatogonia, early prophase spermatocytes, pachytene spermatocytes, and Sertoli cells showing high expression levels.

Transcriptional activation-associated H3K4me3, a specific methylation mark of SETD1B and CFP1, was reported to be at intermediate levels in type A and B spermatogonia and then significantly increased in preleptotene spermatocytes in adult mouse testis (Godmann et al. 2007). In contrast to this study, this methylation was found higher in 10-day-old porcine spermatogonia and remarkably reduced in preleptotene spermatocytes (An et al. 2015). Based on our observation, accumulation of H3K4me3 in the nuclei of spermatogonia, round spermatids, and Sertoli cells may be associated with transcriptional activation and reprogramming of chromatin structure, required for successful spermatogenesis (Sims and Reinberg 2006). Furthermore, given the transient transcriptional repression observed in pachytene spermatocytes (Bettegowda and Wilkinson 2010) as well as in elongating and elongated spermatids (Morgan et al. 2021), it is postulated that reduced H3K4me3 levels in these cells may be associated with transcriptional repression.

The G9A protein interacts with the G9a-like protein (GLP) in order to create H3K9 methylation marks, which are involved in transcriptional repression and heterochromatin reprogramming (Shinkai and Tachibana 2011). Tachibana *et al*., (2007) observed G9A protein in spermatogonia and preleptotene spermatocytes, but not in pachytene spermatocytes, round and elongating spermatids in mice (Tachibana et al. 2007). In contrast, our results demonstrated predominant expression of G9A in pachytene spermatocytes and moderate levels in spermatogonia and round spermatids. This difference may result from using different in techniques and mouse strains.

The absence of G9A led to reduced spermatogenic cell numbers, meiotic arrest, loss of H3K9me2, defective synapse and synaptonemal complex formation, and increased DSBs (Tachibana et al. 2007). Consequently, G9A plays a regulatory role in meiosis-related processes, particularly in spermatocytes. As meiotic defects increase in aging male germ cells during spermatogenesis (Vasco et al. 2012; Yatsenko and Turek 2018), G9A expression was predominantly increased in the spermatogonia, pachytene spermatocytes, and round spermatids of the postpubertal and aged groups possibly to prevent possible meiotic defects and preserve genomic integrity. As in our current finding, H3K9me2 is highly expressed in mouse spermatogonia and preleptotene spermatocytes (Tachibana et al. 2007; Liu et al. 2015). An intense immunostaining of H3K9me2 in elongating and elongated spermatids may be related to transcriptional repression because transcriptional activity is known to cease at the mid-spermiogenesis stage (Lima and Conrad 2017). The observed increase of H3K9me2 levels in the spermatogenic cells with biological aging resulting from enhanced G9A expression might contribute to reduced transcription in aged spermatogenic cells.

Another KMT, SETDB1, has been demonstrated to form H3K9me3 mark in spermatogonial cells, thereby promoting transcriptional silencing in the genes associated with apoptosis (Liu et al. 2017a, b). In mouse testis, SETDB1 protein is predominantly expressed in spermatogonia and preleptotene spermatocytes (Liu et al. 2015), with levels increasing through leptotene and zygotene, and peaking in pachytene and diplotene spermatocytes and round spermatids (Cheng et al. 2021). In porcines, SETDB1 was detected in undifferentiated spermatogonia, primary spermatocytes, Sertoli cells, and intertubular cells, including Leydig cells (Liu et al. 2017a, b). Absence of SETDB1 caused abnormal H3K9me3 accumulation, increased apoptosis, meiotic arrest, abnormal chromosome pairing, and changed gene expression in the spermatogenic cells (Hirota et al. 2018; Li et al. 2020; Cheng et al. 2021). These expression patterns may be associated with a partial transcriptional repression in pachytene stage of primary spermatocytes as well as with strict gene expression control in the remaining spermatogenic cells possibly by creating H3K9me3.

Increased SETDB1 expression in pachytene spermatocytes and round spermatids may result in increased H3K9me3 levels in the elongating and elongated spermatids in the aged testes (Stegeman and Weake 2017). However, Tatehana *et al*., (2020) found reduced H3K9me3 expression in the primary spermatocytes and round spermatids of old mouse testes when compared to the young ones (Tatehana et al. 2020). This finding indicates that the reflection of increased SETDB1 expression on the H3K9me3 levels in spermatogenic cells necessitates developmental progression.

SETD2 is crucial for generating transcriptional activation-related H3K36me3 marks in spermatogenic cells (Zuo et al. 2018). This protein is predominantly expressed in the nuclei of mouse pachytene spermatocytes, round and elongating spermatids as well as in Sertoli cells (Zuo et al. 2018), partially similar to our findings. In addition to accumulating actively transcribing gene regions (Barski et al. 2007), H3K36me3 also contributes to alternative mRNA splicing through regulating splicing-specific regulators, DNA mismatch repair by recruiting hMutSα on chromatin (Luco et al. 2010), and DNA methylation by controlling the actions of DNMT3A and DNMT3B (Wagner and Carpenter 2012). These cellular processes including DNA methylation, are effectively maintained in the aged spermatogenic cells, particularly in spermatogonia and early prophase spermatocytes, probably through increasing H3K36me3 accumulation with SETD2 activity.

Apoptosis-related gene expression is known to increase with aging (Stegeman and Weake 2017). Consistently, the number of cCASP3 positive cells in the intertubular area significantly increased in the aged mouse testis. Increasing apoptosis with aging may result from decreased production of estrogen and luteinizing hormone (LH) hormones (Gunes et al. 2016), which may increase cell susceptibility to apoptotic signals (Joshi and Dighe 2006; Lewis-Wambi and Jordan 2009). However, the number of cCASP3 positive cells in seminiferous tubules of the postnatal testes was highest in the young and prepubertal groups, the potential reason of that case may be to balance cellular contents during early developmental periods.

Importantly, our study contributes to addressing heterogeneity of the aging phenotype in the list of knowledge gaps in biogerontology research that explained in the editorial by Rattan (2024) (Rattan 2024). The present findings offer novel insights into the heterogeneity of testicular aging with regard to KMTs and histone methylation dynamics. An important knowledge gap is how to differentiate whether these changes have adaptive, beneficial or harmful effects on spermatogenic cells in the way of sperm production.

Although spatiotemporal distribution of the KMTs and their target methylation marks were comprehensively evaluated during testicular aging, there are some limitations in this study. While it provides insights into expression levels of various KMTs and associated methylation marks, the current study does not explore the underlying molecular background by which mechanisms these changes affect spermatogenesis. Moreover, it would be beneficial to assess the enzymatic activity of these KMTs as well as demethylases in each spermatogenic cell throughout biological aging.

## Conclusion

In conclusion, the SETD1B, CFP1, G9A, SETDB1, and SETD2 histone lysine methyltransferases and their specific histone methylation marks H3K4me3, H3K9me2, H3K9me3, and H3K36me3 show spatial and temporal expressional differences in the postnatal testes from the young to the aged periods. We found that while relative SETD1B, G9A, SETDB1, and SETD2 protein levels remarkably increase, the CFP1 protein decreases in the aged mouse testes. Similarly, we have detected notable changes in their target methylation marks, including H3K4me3, H3K9me2, H3K9me3, and H3K36me3. Lastly, although we do not observe a change in the transcriptional activity, the apoptosis biomarker cCASP3 expression shows significant increases in the early and aged periods.

In the light of these findings, further investigations are required on the expressional regulation mechanisms of these proteins and the histone methylation marks in spermatogenic cells during biological aging. Furthermore, it is crucial to elucidate the potential impacts of these changes on expression of the genes related to spermatogenesis. Clarification of these issues may facilitate development of novel treatment approaches for mitigating fertility loss accompanying with biological aging.

## Data Availability

The datasets generated during the current study are available from the corresponding author on request.
